# Notes

**Published:** 1895-10

**Authors:** 


### Notes.

The *Dominion Medical Monthly* has united with the Ontario *Medical Journal*. The combination is a strong one and we wish it success.

Dr. J. C. Warren.—The honorary degree of LL. D. has been conferred upon Dr. John Collins Warren by the trustees of Jefferson Medical College.

The *Medcial Messenger* of Fort Madison, Iowa, is the latest addition to medical journals. It is edited by Dr. J. M. Thornber, and though very young, is promising.

Dr. M. C. McGannon, of New York, has been elected and installed Professor of Diseases of Women and Abdominal Surgery in the University College of Medicine, Nashville, Tenn.

During the past month medicine and science have lost some shining lights in the death of Bardeleben, Schimmelbusch, and Pasteur. The loss of the latter following upon that of Tyndall, and more recently of Huxley, creates a gap in the ranks of scientific workers that will probably not be filled during this century.

The St. Louis *Medical Fortnightly* recently made the amusing blunder, in connection with the report of the last meeting of the British Medical Association, in presenting an excellent photograph of Jonathan Hutchinson over the name of G. Watson Cheyne. Mr. Hutchinson's benevolent brown eyes, huge, bulbous nose and Parkhurstian beard are not soon forgotten nor easily confused with the big personality of Mr. Watson Cheyne.

The editor of the *Texas Courier-Record of Medicine* falls under the criticism of being one who has "just enough of learning to misquote." An examination of that rusty old classic, "Pollock's Course of Time," will reveal the origin of the sententious line, "Procrastination is the thief of time," and will relieve Shakespeare of the imputation of the authorship of this antique truism, which has so long graced, in elegant curves and shadings, the pages of the "Spencerian Copy Book."

DR. George Brown, as president of the Alumni Association of the Southern Medical College, has called a meeting to be held in the hall of the college October 18th at 12 M. The following is the interesting program:

Speech of the Hon. W. C. Glenn on the Relations of Law and Medicine.

Speech on behalf of the Faculty, by Dr. Thos. S. Powell.

Speech on behalf of the Alumni, by Dr. John R. Shannon, of Cabaniss, Ga.

BABY FOODS.—The task of raising a baby by hand is a stupendous one and should not be attempted unless imperative. There is no exact substitute for mother's milk, but if Imperial Granum or Mellin's Food are judiciously used rosy, healthy, well-nourished children are the results. While cow's milk with proper dilution is possibly preferable to either of the above, yet the difficulty in preparing it properly and the danger that results if this is not done, serve to give it a secondary rank.

DR. BENJAMIN RUSH.—The Rush Monument Committee of the American Medical Association, are working hard to increase the fund which is to be used for the purpose of erecting a monument to him in Washington. The sum total of the fund, up to September 23d, was $3,357.39. While the energetic and patriotic members of the American Medical Association are thus laboring to secure for Dr. Rush an enduring monument in brass, a literary gentleman and historical student of this city, Mr. Paul L. Ford, is busily at work undermining the eminent doctor's character. According to Mr. Ford, Dr. Rush was a politician of the lower stripe of the present time. His political associates, we are told, were among the trickiest of Philadelphia's tricky politicians, and he got into Congress through a "job," and was not in any wise entitled to sign the Declaration of Independence. This is the point of view of a historical investigator, who has paid a good deal of attention to the contemporary documents. We are not in a position to deny the truth of some of his charges, but the life and work of Dr. Rush, as made known by his writings and his scientific attainments, are, we think, sufficient evidence that he was a man of superior mould, and of a higher character than his critic would admit.—*Medical Record*.

